# Review: roles of mycorrhizal symbioses and associated soil microbiomes in ecological restoration

**DOI:** 10.3389/fmicb.2025.1456041

**Published:** 2025-07-23

**Authors:** Christine Lethielleux-Juge

**Affiliations:** Irrigation NORCO Inc., Québec, QC, Canada

**Keywords:** ecological restoration, mine restoration, mycorrhizal symbioses, soil microbiome, metal-contaminated soils, AMF, ectomycorrhizae

## Abstract

The ecological roles of Arbuscular Mycorrhizal Fungi (AMF) are diverse, providing essential nutrients to host plants, tolerance to stress, and regulation of metabolic pathways, greatly involved in soil C dynamics, unlocking minerals and promoting reactive Fe minerals. Although spores dispersal modes are still not clearly understood, a strong positive relationship exists between intra-and extraradical mycelium at the ecosystem level. AMF are essential in ecosystem restoration by improving soil attributes, above and belowground biodiversity, seedlings survival, growth, and establishment on stressed soils, driving plant succession and preventing plant invasion. AMF inoculants from native and early seral instead of exotics and late seral, consortia instead of few or single species, are more efficient. Plant and AMF communities evolve together after revegetation, fine fescues are among the most resilient species, especially *Festuca rubra*, whose fungal strategies have been recently finely studied. Distinct AMF communities are associated with functionally different plants, which are related to differences in P and C transportomes and genetic variations within the AMF symbiont. Ligneous species react differently to forest soil inoculations according to their arbuscular mycorrhizal symbiosis (AM) or ectomycorrhizal symbiosis (EM) status, and in dual-mycorrhizal plants, costs and benefits are context-dependent, with mycorrhizal switch occurring under various abiotic or biotic factors and resource availability. In mine restoration, root colonization is generally very low during the first year post-reclamation, then increases rapidly before stabilizing. Parallel to plant successions, increased soil parameters, and decreased contaminants, AMF diversity increased and changed, affiliated *Glomus* genera with small spores being completed by *Acaulospora* or *Gigaspora* larger spores under southern climates. A similar recovery period was observed for fungal communities in forest restoration, where ectomycorrhizal mycorrhizal fungi (EMF) species dominate, and diversity increased with time post-revegetation, influenced by edaphic variables and tree species. Under heavy metal (HM) contamination, microorganism classes, enzymes, and AMF efficiency vary with time, soil parameters, restoration treatments, plant species, and levels of soil contamination, with Proteobacteria and Actinobacteria being often predominant. Dual applications of specific microbial and AMF species induced synergistic effects on plant growth and soil resilience. Under other contaminants, several AMF and microbial consortia proved to favorize plant growth and nutrient availability and decrease soil toxicity. New quality indicators to compare rehabilitation studies are proposed.

## 1 Introduction

The consideration of soil microbiome as an indicator of the environmental health of more or less degraded anthropic areas and to measure pedogenesis evolution is very pertinent and has become relatively common in numerous studies. In function of scientific competences and/or ecosystems, arbuscular mycorrhizal (AM) and/or ectomycorrhizal fungi (EMF), total fungi, and/or 16S prokaryotes are considered to evaluate the soil microbial diversity. In this review, we aim to list the efficiency and specificities of those various soil microbial constituents in association with AMF to evaluate the recovering health of anthropic ecosystems. In Section 2, we will successively address the roles of the mycorrhizal symbioses in *ecological restoration* through the general ecological roles of the AMF (2.1), the AM symbiosis in restoration of various ecosystems (2.2), the plant and AMF communities (2.3), and the AM and EM symbioses (2.4). In Section 3, the roles of the AMF, EMF, and plants in *mine restoration* through herbaceous and AMF communities (3.1), as well as in forest environments and EMF communities, are discussed (3.2). In Section 4, the roles of AMF, plants, and *soil microbiome* in the ecological restoration of metal-contaminated soils (4.1) and other ecosystem restoration, emphasizing the importance of parameters standardization, are discussed (4.2). We will conclude with a general assessment emerging from the present reviewing study. Publications of Sections 2 and 3, involving plants, soil, and mycorrhizas, are synthesized in Table 1 and those of Section 4, involving larger microbiome interactions, in Table 2.

**Table 1:** Synthetic results of cited publications involving plant–soil-mycorrhizas consortia in various environmental or experimental contexts.

Soil degradation issues or context of experiment	Degree of plant diversity	Native, natural vs. exotic plants observed or used	Native vs. exotic mycorrhizal inoculum vs. retrieved species	Post-restoration time of observation	Diversity of changes observed and/or results	References
Restored land vs. treeless savana in Venezuela	High	Exotic grasses	Native AMF	25 years	Plant species all mycorrizal	Rosales et al. (1997)
Restored (r) vs. non-restored (nr) forest, Margarita Island, Venezuela	Tropical dry forest plant community	Exotic (r) vs. native (nr) tree species with natural herbaceous	Native *Claroideoglomus*, *Rhizophagus*, Gigasporaceae (r) vs. *Diversispora* and *Funneliformis* (nr)	4 years	Higher AMF species richness in restored areas vs. non-restored areas	Fajardo et al. (2015)
Restored (r) vs. non-restored (nr) landfill, China	Subtropical landfill plant community	Exotic (r) vs. native (nr)	*Glomus*, *Paraglomus*, *Rhizophagus* (n + nr) *Acaulospora*, *Redeckera* (nr) *Scutellospora* (r)	18 years	AMF species more heterogeneous in restored vs. natural sites varying with soil N and CEC	Chen et al. (2018)
Dry tropical forest converted to grassland in Central America	Various species from grasslands, edges and forest habitats	Exotic grasses and natural forest trees	*Gigaspora*, *Scutellospora* ↑ in forest; *Acaulospora*, *Glomus* unchanged	5–15 years	AMF β-diversity ↓; total spore density and α-diversity unchanged	Johnson and Wedin (1997)
Reforestation of a native Hawaiian forest	Reintroduction of native host plants	Native	Native AMF communities altered	30 years	Negative effect of native host plants on AMF communities	Wall et al. (2020)
Various coastal wetlands	Coastal plant communities	Native	Native AMF	Various timelapses	AMF ↑ nutrient uptake, rhizospheric soils, plant resistance to salt and flooding stress	Wang et al. (2022)
Artificial island between Denmark and Sweden	Natural patches of forbs and grasses patches + rare *Salix* and *Hippophaë*	Natural dispersal	Structure of AMF communities compared to neighboring natural island	12 years	AMF richness ↓ due to differential potential of dispersion by migratory birds	Nielsen et al. (2016)
Restored riparian areas in Brazil vs. preserved or degraded sites	Medium	Reintroduction of 10 native tree species including 9 legume trees	Exotic AMF inoculation with OM or rock P fertilization	?	Inoculation ↑ soil aggregation, root colonization, AMF spore number and richness	Pagano et al. (2022)
Serpentine grassland vs. tallgrass prairie	Greenhouse experiment	Two native grasses dominant in both sites	Native and foreign AMF inoculum originating from both sites	13 weeks	Differential responses and competitive abilities of native vs. foreign inoculum	Ji et al. (2010)
Various degraded lands under all latitudes and climates	Various plant biodiversity	Early seral vs. late seral native plant species	Natural, early seral and consortium of AMF species better than exotic, late seral and few or single species	Various timelapses	↑ Soil attributes, above and belowground biodiversity, tree seedlings survival, driving of plant succession and ↓ invasive species	Asmelash et al. (2016)
18 dune restoration projects in US Great Lakes region	Increasing with time	Plantations of native grass *Ammophila breviligulata*	AMF spores abundance compared to reference sites	1–25 years	Plant diversity and AMF spores ↓ in younger vs. older sites	Emery and Rudgers (2010)
Various restoration environments	Various	Exotic Fescues	Amazing AMF root colonization patterns depending on mineral/organic soil type	Various timelapses	Highly resilient grasses which guarantee restoration success	Braun et al. (2020) and Corcoz et al. (2022)
Diverse restoration ecosystems	Increasing plant diversity	Exotic vs. natural species	Natural genetic diversity of AMF evolving with plant species	Various long periods	AMF diversity following plant diversity	Aavik et al. (2021) and Davison et al. (2020)
Post-fire restoration of a tropical forest in Mexico	Six early- + late-successional tree species	Natural successional species	Early- (*Glomus* spp.) and late-successional (*Gigasporaceae*) AMF inoculum	Rapid conversion	Early-seral AMF more efficient to colonize seedlings even for late-seral tree species	Allen et al. (2003)
Various ecological environments	Various forbs, C4, non ruderal vs. ruderal and obligate-mycorrhizal	Exotic or natural	AMF α-diversity ↑ in C4 and non ruderal plants vs. β-diversity ↑ in C4, ruderal and obligate-mycorrhizal plants, AMF diversity ↑ in forbs	Various timelapses	Functionally different plants associate with distinct AMF communities	Davison et al. (2020)
Symbiocosm system, monoaxenic cultures of AMF	*Populus trichocarpa (P.) & Sorghum bicolore (S.)* + different host plants	*In vitro* or monoaxenic cultures	monoaxenic cultures of *Rhizophagus irrigularis*	NA	Specific PO_4_ and NH_4_ transporters on mycelia with *S.* + *P.*, differential regulation, AMF colonization and primary metabolism varying with [P], plant and fungus control nutrients fluxes, homo-and-di-karyotic genetic strains associated with ≠ host species	Calabrese et al. (2019), Kokkoris et al. (2021) and Terry et al. (2023)
Tree plantations in abandoned agricultural field	Two AM (red ash, red maple) and two EM trees (red oak, yellow birch)	Re-introduced trees	Forest soil inoculation from AM or EM dominant stands	1–3 years post-inoculation	+ or − rapid responses for EM trees ≠ after 3 years only yellow birch with red-oak soil still benefit from soil inoculation	St-Denis et al. (2017)
Reclamation of coal surface mine in western Kentucky, US	Natural revegetation	Natural	1st group: *Glomus* sp. soon after reclamation then declined; 2nd group: soon after reclamation then maintained; 3rd group ⊂ larger spores: rare in the first years then ↑	1–5 years	AMF propagules ↑ with time up to 10 species after 5 years	Gould et al. (1996) and Gould and Hendrix (1998)
Reclamation of coal surface mine in Wyoming, US	Natural revegetation	Native *Artemisia tridentata*	Inoculation of native AMF	1 to 15 years (y.)	1–2 y. later, inoculation not efficient ≠ 15 y. later, soil conditions + AMF spores and root mycorrhizal levels similar to adjacent undisturbed soil	Frost et al. (2001)
Diamond mine in Canadian sub-arctic	Natural revegetation	Cyperaceae, grasses and other natural plant species	Natural AMF dominated grasses and DSE species dominated Cyperaceae	1, 3, and 10 years	Low AMF diversity at first increasing with plant diversity; efficient topsoil amendment	Boldt-Burischa and Naeth (2017)
Iron mine tailings restoration in northern Québec and Labrador	Increasing with time	Adapted annual then perennials grasses including *Festuca* and *Phleum* then natural species	Natural AMF genera switching from *Claroideoglomus* to *Rhizophagus*, 2 y. AMF cycle in northern climate	25 years	Beginning of soil pedogenesis, AMF root colonization, AMF species and plant successions	Juge et al. (2021)
Rehabilitation of iron mine sites under Brazilian tropical climate	Increasing with time	Natural vegetation	Mycorrhizal communities evolving toward those of reference sites	1–12 years	AMF spore density and diversity, mycorrhizal colonization, glomalin content and soil pH, texture, SOM and CEC responded positively to the rehabilitation process	Rodríguez-Rodríguez et al. (2021)
	Increasing with time	Natural vegetation	Firstly, half of AMF richness then increased morphotypes mainly *Glomus* sp. and *Acaulospora mellea*	2–3 years	Increased AMF richness, spore number and root colonization	Santiago et al. (2022)
Differential tailings deposition in India	NA	Natural species ⊂ *Homolanthus* sp., *Polyalthia glauca* and *Pandanus* sp.	*Glomus, Acaulospora, Scutellospora* and *Gigaspora* genera retrieved and recommended for rehabilitation programs	?	AMF spores ↑ with *Homolanthus* sp. and *Polyalthia glauca* while root colonization ↑ with *Pandanus* at upper site	Djuuna et al. (2023)
Fly ash ponds in India	NA	Natural plant species ⊂ seedlings of *Eucalyptus tereticornis*	8 morphologically different AMF species and 7 AMF sequence types among *Glomus* and *Archarospora* genera; inoculation with stress adapted *Glomus* and *Scutellospora* consortium + colonized root pieces	?	Inoculation ↑ plant growth, chlorophyll, total P and ↓ Al, Fe, Zn, Cu compared to non-inoculated seedlings	Babu and Reddy (2011)
Cu mine tailings in China	Pot experiment with 4 plant species	2 native *Coreopsis drummondii* and *Pteris vittata +* 2 exotic species *Lolium perenne* and *Trifolium repens*	Inoculation of *Glomus mosseae*	NA	Successful mycorrhizal colonization for all plants tested, ↑ plant yield except for *L. perenne*, ↑ P nutrition, ↓ shoot Cu, As and Cd	Chen et al. (2007)
As contaminated soil in China	Artificial compartmented system	2 exotic species *Lolium perenne* and *Trifolium repens* with roots freely intermingled or separated	Inoculation of *Glomus mosseae*	NA	↑ P nutrition, ↓ root to shoot As translocation and shoot As for both species, mycorrhizas preferentially benefiting clover plants with nutrient acquisition and biomass production	Dong et al. (2008)
Open-pit clay mining substrate ± sand in Eastern China	Experiment with maize planted	Exotic species	AMF *Funneliformis mosseae* inoculation	NA	AMF inoculant ↑ maize growth in clay ± sand, plant biomass, IAA and cytokine levels ↑ in sand-clay, abscisic acid ↓ in sand-clay	Song et al. (2020)
Alkaline gold mine tailing in South Africa	Slow spontaneous plant succession	Native shrub *Dodonaeae viscosa +* grasses *Andropogon eucomus* & *Imperata cylindrica*	Natural AMF colonization from polluted vs. non-polluted sites	30 years	Grasses highly mycorrhiza dependent; AMF colonization ↑ in from non-polluted sites; biomass and survival ↑ with AMF from polluted sites	Orłowska et al. (2011)
Restoration of overburden gold mine in Ontario, Canada	Greenhouse trial with *C. canadensis*, *E. macrophylla*, *F. virginiana*	indigenous plant species	Inoculation with a commercial strain of *R. irregularis* or indigenous AMF community	17 weeks	commercial AMF strain ↑ hyphal and vesicular root colonization; no difference in arbuscules rate in roots nor plant biomasses	Rapai et al. (2016)
Open pit copper and gold mine soils and tailings in British Columbia, CA	2 phases greenhouse experiment with re-introduced successional species	1. Soil conditioned with early-successional *Salix* or late-successional *Picea*; 2. *Picea* and/or *Thuja* growth on soils ± conditioned	AM and/or EM mycorrhizal colonization depending on soil conditioning and tree species	?	Interspecific legacies: − for *Salix*, with pathogen accumulation; neutral to + for *Picea*, with ↑ AM and/or EM colonization and diversity	McMahen et al. (2022)
Iron tailings deposit in China	Natural ecosystem development	Natural succession with *Pinus koraiensis*, *Populus simonii* and *Robinia pseudocacia* trees associated with herbaceous species	Following of EMF communities	5 years	Presence of both trees and herbaceous ↑ soil C, N, P; EMF communities depend on plant species, EMF diversity ↑ with *Robinia* vs. *Pinus* and *Populus* trees	Zhu et al. (2022)

**Table 2:** Synthetic results of cited publications involving plant–soil-mycorrhizas-microbes consortia in various environmental or experimental contexts.

Soil degradation issues or experiment	Degree of plant diversity	Native vs. exotic plant species observed or used	Native vs. exotic mycorrhizal inoculum or present species or root AM colonization	Microorganisms present or inoculated	Time of observation	Diversity of changes and/or results	References
NA	Varying	NA	Native AMF	Diverse	120,000 years of soil chronosequence	Diversity ↑ with short indirect paths rather than many direct interactions	Tylianakis et al. (2018)
Chemical fertilizers and pesticides in various biotic and abiotic agricultural environments	Various	Agricultural species with various AM responsiveness AM-optimized crops	Native and exotic AMF	Various species of biostimulants	A few years depending on pollution degree	Tolerance against various stress, bioremediation of degraded soils, plant-fungal genotype combination	Wu et al. (2022), Begum et al. (2019), Sahraoui Lounes-Hadj (2013) and Berger and Gutjahr (2021)
Review in various environments	Various	Native and exotic plants	Native and exotic AMF	Various microbial interactions	Various	Sink demand and roles of AMF in SOM and C sequestration overlooked	Parihar et al. (2020)
Fe minerals, soil C and AMF relationships in subtropical China	1 plant used as model	Exotic maize in mesocosm greenhouse experiment	Native *Glomus*, *Paraglomus*, *Acaulospora*	Several genera associated with reactive minerals	12 weeks	SOM, CO_2_ emission, reactive Fe and soil C ↑ with AMF; biofilms and mineral hyphal coating	Li et al. (2023)
Heavy Metal (HM) and metal(loid)s contaminated soils	Various	Various	AMF mycorrhizoremediation	Biofertilizers, biostimulants, bioprotectants + AMF associations	Various	Ecological complexity of microbes in the mycorrhizosphere; plants, bacteria, fungi, microfauna, soils and climates dynamics need collaborations between biologists, chemists and physicists; plant-microorganisms-based phytoremediation techniques	Khan (2006), Smith (2002), Jha and Songachan (2022) and Raklami et al. (2022)
HM pollution	Various	Various plants and hyperaccumulation species involving regulation mechanisms against HM stress	AMF, DSE and plant growth promoting endophytic fungi relationships	NA	Various	Plant-fungi coevolution strategies, immune regulation, detoxification transport, balance of host hormones, osmotic regulation, C and N metabolism	Zheng et al. (2023)
Metal-contaminated mine waste in Montana, US, remediated with limed ± topsoil vs. raw tailings ± tilled as controls	Varying with soil treatments	Research plots planted with a seed mixture	AM colonization of 2 grass species ↓ in control and surface-limed plots vs. ↑ in deep-limed and topsoil	Microbial community structure and C-utilization diversity	6 years	Heterotrophic bacteria ↑ under limed and to soil vs. controls; actinomycetes and fungi ↑ in tilled raw tailing; endospores ↑ in topsoil and undisturbed plots; C-utilization ↓↓ in raw tailing, medium in lime and ↑↑ in topsoil and undisturbed plots	Moynahan et al. (2002)
Zn smelters restoration in Pennsylvania, US with low (LC) or high (HC) HM contamination	2 grass species tested	Exotic C4 grass *Sorghastrum nutans* (*S.n.*) and native C3 grass *Deschampsia flexuosa* (*D.f.*)	AMF origin (LC or HC) did not affect root colonization but non-mycorrhizal morphotypes (NMF) varied in *D.f.*	Soil microbial wash	12 weeks	Soil microbial wash ↑ efficiency of AMF from LC but ↓ efficiency of AMF from HC; *S.n.*: high mortality in HC; *D.f.*: biomass unchanged, variation of NMF and shoot Zn	Glassman and Casper (2012)
2 sites under acid-metal spill of a pyrite mine in Spain, reclaimed or not vs. nonpolluted control	?	?	AMF fatty acids (FA) marker analyzed	microbial response explored by PLFAs and ELFAs methods	?	Cu, CD, Zn, pH mostly affected by microbiome structure; stress marker monosat FA ↑ in reclaimed and polluted soil; general fungal marker, AM and Gram+ ↓ with pollution; Gram- ↑ in polluted soil; ELFA method sensitive	Hinojosa et al. (2005)
Revegetated areas around a Pb/Zn smelter under subtropical climate in China vs. control sites	Medium	Native species *Paulownia fortunei + Cynodon dactylon* and exotic heavy metal-tolerant species	AMF PFLA marker	Microbiome structure explored by PLFAs and enzymes	2, 3 and 5 years	Revegetation did not change PFLAs and enzyme profiles; Gram-, AMF, total fungi, actinomycetes, algae, protozoa, fung/bact, FA and enzymes + soil N, pH and porosity ↑ with time post-revegetation	Zhang et al. (2006)
Dolomite-amended polymetallic contaminated tailings in Arizona, US	One species tested	Native perennial grass	NA	Consortium of N_2_ -fixing microbial endophytes and waste compost	45 days	Dolomite + N + seed-coated endophytes ↑ C and yields; compost ↓ shoot and root metal contents; endophytes ↑ foliar Cd, Co, Mn and Pb	Creamer et al. (2023)
Ultramafic soils in Albania	4 species tested	Native Ni hyperaccumulating plants: *Noccaea o.*, *Odontarrhena smolikana*, *O. rigida*, *O. chalcidica*	NA	Potential functions of rhizosphere-associated bacteria explored	NA	Proteobacteria, Actinobacteria and Acidobacteria dominant; Proteobacteria influenced by CEC; genes belonging to amino acid, lipid and carbohydrate metabolisms identified with predicted metagenomes	Lopez et al. (2019)
Deactivated Fe mine exploitation in Brazil	Medium	Comparison of revegetation (RV) vs. natural (NT) site at proximity	NA	N_2_-fixing microorganisms, actinobacteria (actinob.) and other groups of soil microbes explored by DGGE profiles	1–10 years	Euryarchaeota, Thaumarchaeota, Proteob., Actinob., Acidob., Verrucomicrobia: most abundant groups; Proteob., Actinob. ↑ in RV; Acidob. and Verrucomicrobia ↑ in NT but RV and NT mostly share the same main microbiome; P, pH, particle density ↑ in RV; Fe, Ca, SOM and clays ↑ in NT	Cardoso et al. (2020)
Gold mining waste rock dumps rehabilitation in Burkina Faso	One species studied	*Senegalia senegal* seedlings grown on 3 substrate types then out-planted on waste rocks	Inoculation with native isolate of *Rhizophagus aggregatus* (*R.a.*) or the commercial isolate of *R. irregularis* (*R.i.*)	Inoculation with *Mesorhizobium plurifarium* (M*.p.*) strain	3, 12 and 72 months	Manure-enriched substrates ↓ nodulation and AM colonization but ↑ growth; highest plant growth with *R.a.* ± *R.i.* or *M.p.*; *R.a.* + *M.p. or R.i. ↑* root colonization in un-amended substrates; plant growth and survival ↓ under high manure and *↑* under un-or low-amended substrate due to better nodulation and mycorrhization in field	Yonli et al. (2022)
Abandoned extreme antimony Sb tailings dump vs. adjacent normal soils in China	One plant species sampled	Native *Bidens bipinnata*	NA	Root-associated bacteria	NA	Rhizosphere microbial diversity ↑ in tailing dump ≠ in adjacent soils, related to soil C, N, Sb and As; N-fixing, P-solubilizing, Sb-and As-oxidating genera *↑* in tailings	Xiao et al. (2019)
Mycoremediated dry olive residue (MDOR) in metal-polluted soil?	NA	Mesocosm experiment with mycoremediated dry olive residue	Inoculation with *Funneliformis mosseae*	Inoculation with *Penicillium chrysogenum* or *Chondrosterum purpureum*	30 and 60 days	Changes in soil labile C and N fractions; PFLAs ↑ for bacteria, actinobacteria, Gram-and Gram+; MDOR ↑ fungi and AMF; MDOR + *F.mossae* ↑ AMF root and soil colonization	García-Sánchez et al. (2019)
Coal mining wastes rehabilitation in China	↑ in planted than in naturally regenerated areas	Natural revegetation vs. plantation of native species	NA	Bacterial community structure and diversity described	14 years	Greater impact of soil pH, C, N, SOM, moisture and bulk density on bacterial community structure and diversity	Liu et al. (2019)
	one plant species tested	two-compartments microcosms with *Medicago sativa* (*M.s.*)	Inoculation of a *Glomus mosseae* strain	Inoculation of a P-solubilizing bacteria (PSB) strain	60 days	available P, above and underground biomass of *M.S.* ↑ with AMF-PSB treatments in root and hyphae compartments	Bi et al. (2019)
Petroleum contaminated soils	Medium	4 natural plant species growing in non-contaminated vs. contaminated sediments	AMF assemblages associated with root rhizosphere described	Repeated inoculations with a 10 isolates consortium of Proteobacteria	16 weeks	Bacterial inoculation shifted AMF communities, ↑ plant biomass, ↓ petroleum hydrocarbons but contaminated substrate had a stronger influence on AMF communities	Dagher et al. (2020)
	NA	NA	*R. irregularis in vitro* exposed to a polycyclic aromatic hydrocarbon	NA	NA	Fungal triacylglycerols metabolism involved in hydrocarbon translocation and degradation	Calonne et al. (2014)
Mediterranean salt marsh in Southeast Spain, coastal ecosystems	Medium	Effect of a spatial salinity gradient on the native species *Inula crithmoides*	AMF root colonization explored	Microbial and biochemical rhizosphere properties explored	NA	No difference in soluble C, carbohydrates fractions or microbial biomass C values; dehydrogenase activity, hydrolases and AM colonization ↑ in lower soil salinity; bioactive SOM not related to salinity	Caravaca et al. (2005)
Various coastal ecosystems	High	4 natural coastal communities: sand dunes, marshes, mangroves and forests-shrublands	Ecology of mycorrhizae, N fixers and endophytes in restoration reviewed	Rhizosphere microbes and pathogens reviewed	Various	Microbial symbionts largely responsible for coastal plants establishment, growth, competitive ability, stress tolerance and modulation of biogeochemical cycling	Farrer et al. (2022)
Open coal mine	7 to 13–17 plant taxas vs. 19–20 in forests	Natural progressive revegetation of several sites compared to 2 forests in proximity	NA	General Indicator of Soil Quality (GISQ) tested with 3 sub-indicators	1–20 years	Soil pH, bulk density, macro-aggregates ↑ in 1-year site; SOM, N, biogenic aggregates ↑ in older sites; GISQ ↓ in 1-year site (0.1–0.3), medium in 16-20-years sites (0.4–0.7) and ↑ in forests (0.4–1.0)	Domínguez-Haydar et al. (2019)
Rehabilitated iron ore mine in Brazil	Rehabilitation indicators of vegetation structure and community diversity	Natural rehabilitating chronosequence	Easily extractible and total glomalin content	Richness and Shannon diversity of microorganisms communities, metabolic quotient, N and C in microbial biomass	2, 3, and 6 years	32 environmental variables of ecological processes, vegetation structure and community integrated by multivariate ordination; case-specific environmental indicators defined for more rapid assessment of mineland rehabilitation	Gastauer et al. (2020)

## 2 Mycorrhizal symbioses in ecological restoration

### 2.1 General ecological roles of arbuscular mycorrhizal fungi (AMF)

**Figure 1:**
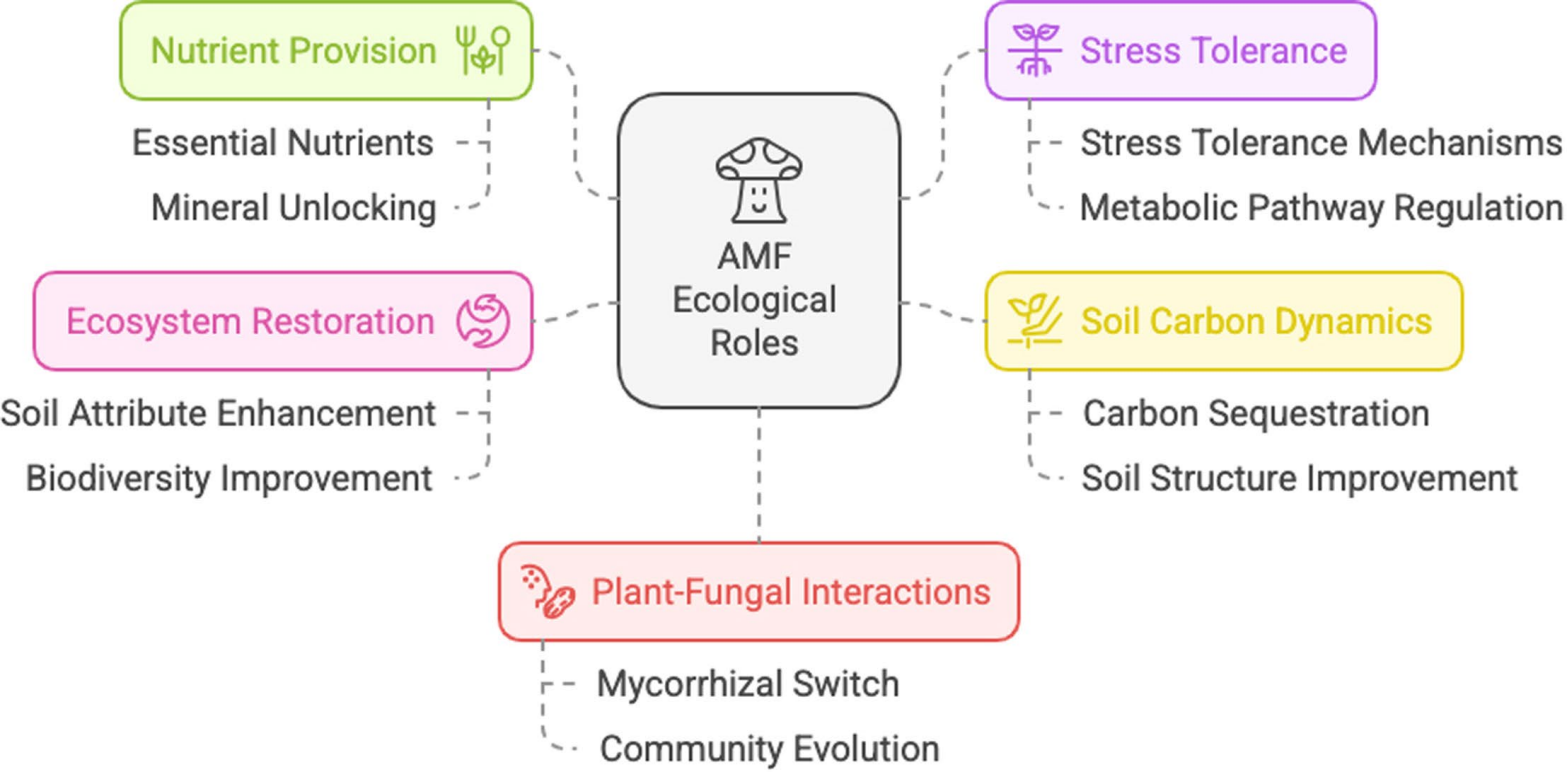
Schematic representation of the main ecological roles of arbuscular mycorrhizal fungi. Created by Napkin.

The ecological roles of AMF are diverse, including indirect pathways, through changes in plant and soil microbial community composition, direct pathways, i.e., effects on host physiology and resource capture, and direct mycelium effects (reviewed by Rillig, 2004) (Figure 1).

Along a 120,000-year soil chronosequence, Tylianakis et al. (2018) found that network assembly and disassembly of AMF communities were symmetrical, with plant and AMF species that had short indirect paths to others in the community rather than many direct interaction partners being better capable to attract new interaction partners and, in the case of AMF species, to retain existing interactions with plants during retrogression. Those model simulations showed that these non-random patterns of attachment and detachment promote the nestedness of the networks, having implications for predicting extinction sequences, identifying focal points for invasions, and suggesting trajectories for restoration.

In the present context of climate change and soil pollution due to malpractices, such as the excessive use of chemical fertilizers and pesticides in agriculture, the use of AMF as biofertilizers should be favored for enhancing crop productivity (Wu et al., 2022) and providing tolerance to host plants against various stressful situations such as salinity, drought, metals, and extreme temperatures. Indeed, AMF provide essential plant inorganic nutrients to host plants and may assist host plants in the upregulation of tolerance mechanisms and prevent the downregulation of key metabolic pathways (reviewed by Begum et al., 2019).

Furthermore, since mycorrhizal symbioses, and specifically AM symbiosis, play several important roles in C biosequestration, nutrient cycling, plant biodiversity, and productivity of natural and agricultural ecosystems, they appear as a key ecosystem service provided by nature to human society, representing a major challenge, not only for low input sustainable agriculture but also for the management and the remediation of degraded soils (reviewed by Sahraoui Lounes-Hadj, 2013).

Several studies established that AMF contribute predominantly to soil organic matter (SOM) and C sequestration by creating a sink demand for plant C and distributing it to belowground hyphal biomass (reviewed by Parihar et al., 2020). According to previous authors, however, despite the increase of net primary productivity and additional photosynthetic fixed C in the soil through extraradical hyphae and glomalin-related soil protein, the role of AMF in C cycling and climate change models is largely overlooked, and the AMF buffering mechanism against elevated CO_2_ should be considered only after including their potential interaction with other microbes and associated mineral nutrients, such as N cycling.

For example, a thin coating of Fe minerals was recently observed on the surface of AMF hyphae, illustrating the close physical association between fungal hyphae and soil Fe minerals, and *Glomus* genus being positively correlated with reactive Fe minerals while *Paraglomus* and *Acaulospora* being negatively correlated (Li et al., 2023). In this study, the presence of roots and AMF, particularly when combined with litter addition, also enhanced several critical soil bacterial genera associated with reactive minerals in soils, such that root exudates and AMF not only stimulate the decomposition of litter and soil organic carbon (SOC) and promote the production of CO_2_ emission but also seem to drive soil C persistence by unlocking mineral elements and promoting the formation of reactive minerals.

Despite an increasing global understanding of AMF diversity, distribution, and prevalence in different biomes, the main dispersal mechanisms of these organisms have been largely ignored. The available data on AMF dispersal originate mostly from North America and temperate ecosystems, from biotic dispersal agents (small mammals) and AMF spores as propagule type. At the same time, much lesser evidence exists from South American, Asian, and African tropical systems and other dispersers such as large-bodied birds and mammals and non-spore propagule types (reviewed by Paz et al., 2021). Despite the data being too scarce to draw firm conclusions, previous authors found no strong evidence that spore size varies across dispersal agents but that wind and large animals seem to be more efficient dispersers.

To understand and quantify the impacts of AMF on the ecosystem functioning, relationships between AMF abundance and community composition in soil and plant roots were explored across a dune grassland plant community by Barcelo et al. (2020), highlighting a strong positive relationship between the total length of roots colonized by AMF and the amount of NLFA 16:1ω5 in the soil, thus providing the first field-based evidence of proportional biomass allocation between intra-and extraradical AMF mycelium at ecosystem level. This suggests that this phenomenon is made possible by compensatory colonization strategies of individual fungal species and opens the possibility of using AMF total root colonization as a proxy for soil AMF abundances.

### 2.2 Arbuscular mycorrhizal symbiosis (AM) in restoration of various ecosystems

Numerous studies have described the variations in AMF diversity and/or the efficiency of AMF inoculation following operations of ecological restoration in diverse specific ecosystems (Table 1).

In Venezuela, 25 years after the natural colonization of restored degraded land by native plant species, all species studied were mycorrhizal, suggesting to the authors that the restoration program in those degraded areas should take mycorrhizae into account, reintroducing them or manipulating the soils to increase the mycorrhizal inoculum (Rosales et al., 1997). Even with similar root colonization rates, restoration programs in a tropical dry forest at Margarita Island promoted higher AMF species richness and diversity than non-restored controls after 4 years, *Claroideoglomus etunicatum* and *Rhizophagus intraradices* being found in all plots, while *Diversispora spurca* and *Funneliformis geosporum* only in non-restored plots and Gigasporaceae, a family associated with little disturbed sites, commonly observed in restored soils (Fajardo et al., 2015).

In a subtropical landfill in China, Chen et al. (2018) further observed a more heterogeneous AMF community in restored sites than in natural sites 18 years later, with the structure of the AMF communities being influenced by soil N and cation exchange capacity (CEC). *Glomus*, *Paraglomus*, and *Rhizophagus* genera were commonly found at all sites, *Acaulospora* and *Redeckera* exclusively at natural sites, and *Scutellospora* only at the restored site, so that AMF species richness was lower while diversity was higher in the restored site. Since the AMF community at restored sites clearly deviated from that at natural sites, the authors concluded that restoration practice was certainly inadequate. However, since AMF communities are specific to plant species, it seems normal that AMF populations change after the restoration program; they will evolve with time and ecological successions (Juge et al., 2021; see also Section 2.3).

Indeed, through the conversion of a dry tropical forest to grassland in Central America, Johnson and Wedin (1997) found that although the beta diversity of AMF spore communities was lower in the grassland than in forest plots, where *Gigaspora* and *Scutellospora* genera were more present, total spore density and alpha diversity of AMF spore communities—10–18 species mainly belonging to *Acaulospora* and *Glomus* genera—were not altered by wildfires and grass invasion, suggesting that regeneration of forest plant species in those sustainable grasslands may not be heavily constrained by the lack of mycorrhizal symbionts. By contrast, three decades after the reforestation of a native Hawaiian forest did not lead to the reassembly of AMF communities associated with remnant primary forests, inducing potential negative effects on the recruitment of native host plants (Wall et al., 2020). The latest study suggests that the natural cycle of plant ecological successions was not sufficiently respected to progressively reconstruct soil AMF and microbial communities. Since the quality of restoration is not determined by the total diversity but by the capacity of the ecosystem to withstand disturbances to maintain or correct a trajectory, allowing its resilience, co-occurrence networks would probably represent a better indicator than beta diversity.

In coastal wetland environments, AMF are distributed worldwide and are mainly limited by flooding, hypoxia, soil pH, salinity, and host plant identification. In such environments, the AM symbiosis promotes nutrient uptake of host plants, improves the characteristics of rhizospheric soil, and enhances plant resistance to salt and flooding stress (reviewed by Wang et al., 2022). In a newly constructed artificial island between Denmark and Sweden, the AMF Operational Taxonomic Unit (OTU) richness was significantly lower than on a neighboring natural island, indicating that the colonizing AMF must have been restricted by limited dispersal, likely assisted by migratory birds, so that the AMF communities colonizing the new island appeared to be a non-random subset of communities on the natural and much older neighboring island (Nielsen et al., 2016).

In restored riparian plantations in Brazil, inoculation with AMF seemed to guarantee a successful restoration as, in the inoculated restored fields, higher soil aggregation, root colonization, spore number, and richness of AMF were found, compared to the degraded ones, although the undisturbed site presented the highest values (Pagano et al., 2022). Ji et al. (2010) further investigated functional differences among AMF natural communities between a serpentine grassland and a tallgrass prairie, using two grasses common to both systems in a greenhouse experiment. Since the two grasses responded differently to native and foreign AMF, the use of foreign inoculum in restoration could change the relative performance and potentially the competitive abilities of co-occurring plant species, suggesting that a particular AMF community may be better matched ecologically to its local habitat than communities taken from other locations.

Globally, abundant scientific evidence demonstrated that AMF improved the restoration success of degraded lands by improving soil attributes, above and belowground biodiversity, tree/shrub seedlings survival, and growth and establishment on water and nutrients poor soils, as well as driving plant succession and preventing invasion of plant species. Meanwhile, as degraded lands harbor low levels of infective AMF abundance and diversity, the successful restoration of infective AMF can potentially improve the success of land restoration, and better AMF inoculation effects were observed when inocula are composed of native fungi instead of exotics, early seral instead of late seral fungi, and consortia instead of few or single species (reviewed by Asmelash et al., 2016).

### 2.3 Plant and AMF communities in ecological restoration

Several studies have explored the diversity and successions of plant communities, either reintroduced or natural, associated with AMF in ecological restoration (Table 1).

Ecological restoration operations through diverse ecosystems and climates generally showed an increase of plant and AMF diversity with years post-restoration, such as in dune restoration in the Great Lakes region in the US (Emery and Rudgers, 2010) or iron mine tailings in Canada (Juge et al., 2021). The joint effect of host plant genetic diversity and AMF communities on restoration success was reviewed by Aavik et al. (2021). Indeed, since plant functional groups associate with distinct AMF communities (Davison et al., 2020), specific plant species with high mycorrhizal dependency, associated with specific AMF species, can make the difference in ecological restoration success, such as the choice of plant species is a key to guarantee the success of the operations (Juge et al., 2021).

Under the northern environment, agronomic methods consisting of reintroducing seed mixtures of fast-growing cereals favoring the installation of perennial gramineous, then relying on natural successions for the reinstallation of native and ligneous species, have been proved to be successful, parallel to natural succession in AMF species (Juge et al., 2021). Among those efficacious perennial gramineous, fine fescues are the more resilient ones (reviewed by Braun et al., 2020). Corcoz et al. (2022) finely explored the changes in AMF colonization patterns of *Festuca rubra*, one of the dominant species in mountain natural grasslands, by establishing mycorrhizal maps permitting a deep scanning of root colonization and real positioning of AMF structures. The highest overall colonization was found in organic conditions, while low-mineral organic conditions induced a clear separation of colonization strategy in different parts of the roots, and mineral treatment restricted AMF development, which maintained colonization by a resistance conditions strategy. The presence of arbuscules and vesicles in the same root area indicated a continuous alternate of fungal strategy, from storage to enhanced transfer of nutrients.

In a tropical forest in Mexico undergoing rapid conversion to early successional forest because of increased wildfire, Allen et al. (2003) tested the responses of six early-and late-successional tree species using early-and late-successional AMF inoculum. All the tree species had the greatest growth response to early-seral inoculum, dominated by small-spored *Glomus* spp., while under late-seral inoculum, containing high densities of large-spored *Gigasporaceae*, two tree species were smallest and the four other species had intermediate growth. The uninoculated plants became infected by residual inoculum in the burned experimental site within 3 months of transplanting, yet mycorrhizal responses persisted. Mature forest trees may withstand the C drain from *Gigasporaceae* better than establishing seedlings, so the growth patterns observed with inoculum source are consistent with a rapidly growing successional forest, followed by slower-growing mature forest, suggesting that early-seral AMF should be used when seedlings are inoculated for restoration, even for late-seral tree species.

Indeed, the benefits of the AM symbiosis between plants and fungi are modulated by the functional characteristics of both partners, but it is more confusing to what extent functionally distinct groups of plants naturally associate with different AMF. By re-analyzing high-throughput sequencing datasets describing AMF communities associating with plant individuals and examining how root-associating AMF communities varied among plants with different growth forms, photosynthetic pathways, CSR (competitor, stress-tolerator, and ruderal) strategies, mycorrhizal statuses, and N-fixing status, Davison et al. (2020) observed that grasses, C4, and non-ruderal plants were characterized by high AMF alpha diversity, while C4, ruderal, and obligately mycorrhizal plants were characterized by high beta diversity. Therefore, AMF diversity was higher among forbs than other plant growth forms, and putatively ruderal (previously cultured) AMF were disproportionately associated with forbs and ruderal plants, confirming that associated AMF communities constitute an important component of plant ecological strategies and that functionally different plants associate with distinct AMF communities.

Aavik et al. (2021) further reviewed the relationships between the composition of the AMF community and the genetic diversity of the host plant population, considering that in ecological restoration, one of the reasons for the failure to stop biodiversity decline could lie in overlooking the importance of improving the aboveground ecosystem complexity, including the recovery of interaction networks with co-adapted plant species. Since the genetic diversity within a host plant population can have a significant effect on the ability of the host plant to benefit from mycorrhizal associations, they suggest that new genomic approaches, such as community genetics and landscape genomics, should be applied to shed light on this aspect of plant–fungal interactions and be incorporated in restoration planning.

In an agricultural context, the ability of plants to react to AM with changes in morphology and/or performance in terms of yield, called ‘AM responsiveness’, depends on the plant–fungal genotype combination and the abiotic and biotic environment, the genetics and mechanistic remaining still mainly unknown, such that a molecular understanding of AM responsiveness is a key for enabling rational application of AM in agriculture, for example, through targeted breeding of AM-optimized crops (reviewed by Berger and Gutjahr, 2021).

At the physiological level, as phosphate is a nutrient and a regulator of nutrient exchanges in AM symbiosis, Calabrese et al. (2019) investigated the effect of P availability to extraradical mycelium (ERM) on different plant and fungus transcriptomes and metabolomes in a symbiocosm system formed by *Populus trichocarpa*, *Sorghum bicolor*, and *Rhizophagus irregularis*. Transportome analysis on the ERM and intraradical mycelium of *R. irregulare* revealed that mycorrhizal symbiosis induces the expression of specific PO_4_ and NH_4_ transporters in both plants. They identified new AM-inducible transporters and showed that a subset of PO_4_ transporters is regulated independently of symbiotic nutrient exchange and that the fungal transportome was not similarly regulated in the two host plant species according to P availability. Mirroring this effect, many plant carbohydrate transporters were downregulated in *P. trichocarpa* mycorrhizal root tissue. Metabolome analysis further revealed that AM root colonization led to a modification of root primary metabolism under low and high P availability and a decrease in primary metabolite pools. Moreover, the downregulation of the sucrose transporters suggests that the plant limits carbohydrate long-distance transport and that by simultaneous uptake/reuptake of nutrients from the apoplast at the biotrophic interface, plant and fungus are both able to control reciprocal nutrient fluxes. Since we also know that different homo-or di-karyotic genetic strains of *R. irregularis* are associated with different host species (Kokkoris et al., 2021), which influences plant growth response (Terry et al., 2023), it seems that we are not at the end of further big discoveries to understand these unique and fascinating symbiotic functional exchanges between the two communities of symbionts, both at physiological, genetic, and ecosystem levels.

### 2.4 AM and EM symbioses in ecological restoration

Together with the AM symbiosis, the most ancient and widespread throughout the planet, EM symbiosis plays an important role in the restoration of forest ecosystems (Table 1).

In an abandoned agricultural field restored by tree plantations, St-Denis et al. (2017), by testing the effect of adding small amounts of forest soil on the survival, growth, and rates of mycorrhizal fungal colonization of two AM and two EM trees, observed that EM tree species responded, positively or negatively, to forest soil inoculation, the majority of the effects being observed the first year. Moreover, after three seasons of growth, only yellow birch seedlings that had received non-sterilized red oak soil still benefited from soil inoculation, indicating species-specific effects of inoculation on long-term benefits of tree survival and growth under nutrients-limited soil.

Moreover, several ligneous species benefit and take advantage of the two mycorrhizal symbioses. A recent review explored those dual-mycorrhizal plants, which constitute powerful model plant–fungal systems to better understand mycorrhizal symbioses without confounding host effects (Teste et al., 2020). The authors identified 89 genera within 32 families of confirmed dual-mycorrhizal plants based on observing arbuscules or coils for AM status and Hartig net or similar structures for EM status within the same plant species. The cost and benefits of dual-mycorrhizal status appeared to be context-dependent, particularly with respect to the life stage of the host plant, and mycorrhizal switching occurs under a wide range of abiotic and biotic factors, including soil moisture and nutrient status. Furthermore, the endophytic presence of EMF in AMF plants is currently observed, suggesting tripartite interactions through ecological successions (Authier et al., 2022).

Since plant–mycorrhizal interactions mediate plant community coexistence by altering resource demand, the influence of mycorrhizal fungi on plant biodiversity likely depends on the strength of the symbiosis between plants and fungi, differential plant growth responses to mycorrhizal inoculation, and rate of nutrients transfer from fungus to plant. Jiang et al. (2017) developed a mechanistic resource competition model that explicitly included plant–mycorrhizal symbioses. It confirmed that plant–mycorrhizal interactions shape plant coexistence patterns by creating a tradeoff in resource competition, notably caused by differential payback in the C resources that plants invested in the fungal symbiosis and/or by the stoichiometric constraints on plants that required additional resources to sustain growth, resource availability, and variation in plant–mycorrhizal interactions acting in concert to drive plant coexistence patterns.

## 3 AMF, EMF, and plants in mine restoration

Mining exploitation generates tailings and/or large desertic disturbed areas, which must be rehabilitated. Since toxicity levels of heavy metals or other contaminants depend on each type of extracted ore, restoration operations must be adapted to each specific ecosystem and substrate composition. For example, plant and mycorrhizae interactions are as numerous as mining contexts (Table 1).

### 3.1 Herbaceous species and AMF communities in mine restoration

During reclamation of coal surface mine sites in western Kentucky, US, Gould et al. (1996) showed that at the seeding stage and soon after, AMF propagules and spores were low, and root colonization was absent, which then increased rapidly during the first 2 years following reclamation before stabilizing, and SOC and Ca were positively correlated with AMF propagules and spores densities. A high proportion of one group of spore species, mainly *Gl. microcarpum*, *Gl. Aggregatum*, and *Gl. fasciculatum*, was found early after reclamation and then declined; a second group appeared soon after reclamation and maintained a relatively constant proportion; and a third group, notably those with larger spores, was rare for a few years after reclamation but increased with time, such that species richness was low soon after reclamation, rose slowly and erratically over 5 years, and then stabilized at approximately 10 species (Gould and Hendrix, 1998). Moreover, in a severely disturbed reclamation site of a similar coal surface mine in Wyoming, US, inoculation of native AMF did not improve the growth of *Artemisia tridentata* 1–2 years later, while after 15 years, soil conditions, with 1% increase in SOM content, improvement in soil structure, and decreases in amounts of soluble salts and Na, became more similar to those before disturbance, with much greater root mycorrhizal levels and AMF spore density not significantly different from adjacent undisturbed native soil (Frost et al., 2001).

Similarly, under northern boreal forest environment, where mycorrhizae are considered drivers of ecosystem processes (Read et al., 2004) and which is rich in AMF taxons when herbs are present (Öpik et al., 2008), at a diamond mine in the Canadian sub-arctic, Boldt-Burischa and Naeth (2017) observed that on reclamation substrates, natural colonization of vegetation-free sites with AMF spores was very low but that fungal spore quantity and diversity was significantly accelerated by the establishment of vegetation. DSE dominated Cyperaceae on the native site, whereas AMF dominated grasses on the reclamation site, and topsoil amendment was the most effective for fungal colonization on reclamation substrates. We observed a similar early time course of AM installation following plant species diversification during 25 years of restoration of iron mine tailings in northern Québec and Labrador, with the main AMF genera switching, after 5 years, from *Claroideoglomus*, also found in the boreal forest at proximity, to *Rhizophagus*, which seemed more adapted to the reintroduced perennial herbaceous, mainly *Festuca* and *Phleum* genera (Juge et al., 2021).

Under Brazilian tropical climate, Rodríguez-Rodríguez et al. (2021) also showed that AMF spore density and diversity, mycorrhizal colonization, and glomalin contents related to soil pH, texture, SOM, and CEC responded positively to the rehabilitation processes of three iron mining sites. Over time, mycorrhizal communities of rehabilitated areas became more similar to reference sites, and a decrease in the easily extractable glomalin/total extractable glomalin ratio was observed, showing the resilience of native mycorrhizal communities and the success of the rehabilitation actions. Moreover, under the same climate and iron mining sites, Santiago et al. (2022) observed that AMF species richness was reduced by approximately 50% after mining activity, followed by an increase in spores number, root colonization, and AMF species after the initial rehabilitation operations, and *Glomus* sp. and *Acaulospora mellea* are the most frequent morphotypes.

Within tailings deposition areas under low total soil N and P, low to high organic C, and pH 6–6.7 in India, Djuuna et al. (2023) counted 2–20 AM spores/10 g soil and 17–90% of root colonization depending on plant species. The highest number of AMF spores was found in the rhizosphere of *Homalanthus* sp. and *Polyalthia glauca*, whereas the highest percentage of root colonization was observed under *Pandanus* sp. at the upper site. Thus, the highest number of spores did not correspond to the highest percentage of infected roots. Four genera of AM fungi, namely, *Glomus*, *Acaulospora*, *Scutellospora*, and *Gigaspora*, thriving in those tailings areas, are recommended for rehabilitation programs in this region.

Moreover, under Cu mine tailings in China, Chen et al. (2007), by testing two native plant species, namely, *Coreopsis drummondii* and *Pteris vittata*, as well as *Lolium perenne* and *Trifolium repens* associated or not with the AMF *Glomus mosseae*, found that symbiotic associations successfully settled between *Gl. mosseae* and all plants tested. The mycorrhizal colonization markedly increased plant yield except for *L. perenne* and that the beneficial impacts of mycorrhizal colonization on plant growth could be largely explained by improved P nutrition and decreased shoot Cu, As, and Cd concentrations. Dong et al. (2008) further investigated the influence of *Gl. mosseae* inoculation on plant growth, As uptake, P nutrition, and plant competition between *T. repens* and *L. perenne*, grown together in an As contaminated soil, with their roots freely intermingled or separated through a compartimented system. Mycorrhizal inoculation improved P nutrition and markedly decreased root-to-shoot As translocation and shoot As concentrations for both plant species. Mycorrhizas affected the competition between the two plant species, preferentially benefiting the clover plants in terms of nutrient acquisition and biomass production.

In open-pit clay mining areas on grassland in Eastern China, Song et al. (2020) also observed that AMF promoted plant growth in the mining-associated clay, mixed or not with the same proportion of sand. Indeed, the above-and underground biomass of maize in sand-clay soil were higher than in clay soil, the IAA and cytokinin levels in sand-clay and topsoil were higher than in clay, while abscisic acid levels were lower, confirming that AMF inoculation could significantly improve maize biomass and enhance its stress resistance and that soil type and AMF inoculation had the most direct impact on maize growth.

In an alkaline gold mine tailings in South Africa characterized by a slow spontaneous plant succession and colonized by the shrub *Dodonaea viscosa* and the grasses *Andropogon eucomus* and *Imperata cylindrica*, Orłowska et al. (2011) observed that both grasses were highly mycorrhiza-dependent and the presence of mycorrhizal colonization significantly increased their biomass and survival rates. Although the colonization rate with native AMF was lower than with AMF from non-polluted sites, they were more vital and more effective in promoting plant growth, suggesting that the AMF originating from the gold tailing were better adapted to the tailings conditions. However, through the restoration of an overburden gold mine in Ontario, Canada, Rapai et al. (2016), during a 17-week greenhouse trial, observed that soil inoculation with a commercial strain of *R. irregularis* resulted in a greater hyphal and vesicular root colonization than with the indigenous AMF community isolated from revegetated mine rock spoil piles, while no significant variation in arbuscule colonization of *C. canadensis*, *E. macrophylla*, and *F. virginiana* plant community and similar increases in plant biomasses were observed between the two inoculants.

More generally, concerning AMF inoculation, in a global meta-analysis of 193 independent outcomes to understand the overall effect of AMF as a restoration tool and to evaluate sources of variation on its effects on plant development in mining degraded areas, De Moura et al. (2022) concluded that the use of AMF is needed to increase and accelerate the restoration effectiveness of natural processes and ecosystem services in mined areas under restoration, through the increase of water and nutrient uptake by the host plants, therefore decreasing plant stress and improving habitat development. Their results emphasized the great potential of AMF to stimulate plant growth in those degraded areas, with stronger effects if associated with the utilization of SOM and other soil microorganisms. Although most AMF studies are concentrated in temperate regions, AMF inoculum promoted greater plant development on mined substrates regardless of the region, fungi type, or origin. Inoculation also improved plant growth in materials of difficult restoration, such as overburden and tailings, but the AMF effects depended upon the exploited mineral type.

In the northern iron mining environment, we observed that the reintroduced plant communities, then spontaneous perennial plant species, became naturally fully colonized by AMF only 3 years after restoration operations (Juge et al., 2021). However, under more toxic or more degraded environmental areas, such as in the above studies under an overburdened gold mining environment (Rapai et al., 2016), or under temperate or tropical climates such as in China (Song et al., 2020) and India (Babu et al., 2011; Djuuna et al., 2023), the re-introduction of AMF species, with a preference for locally adapted inoculants, has been proved to be useful to accelerate the reinstallation of AM soil network communities (De Moura et al., 2022).

**Figure 2:**
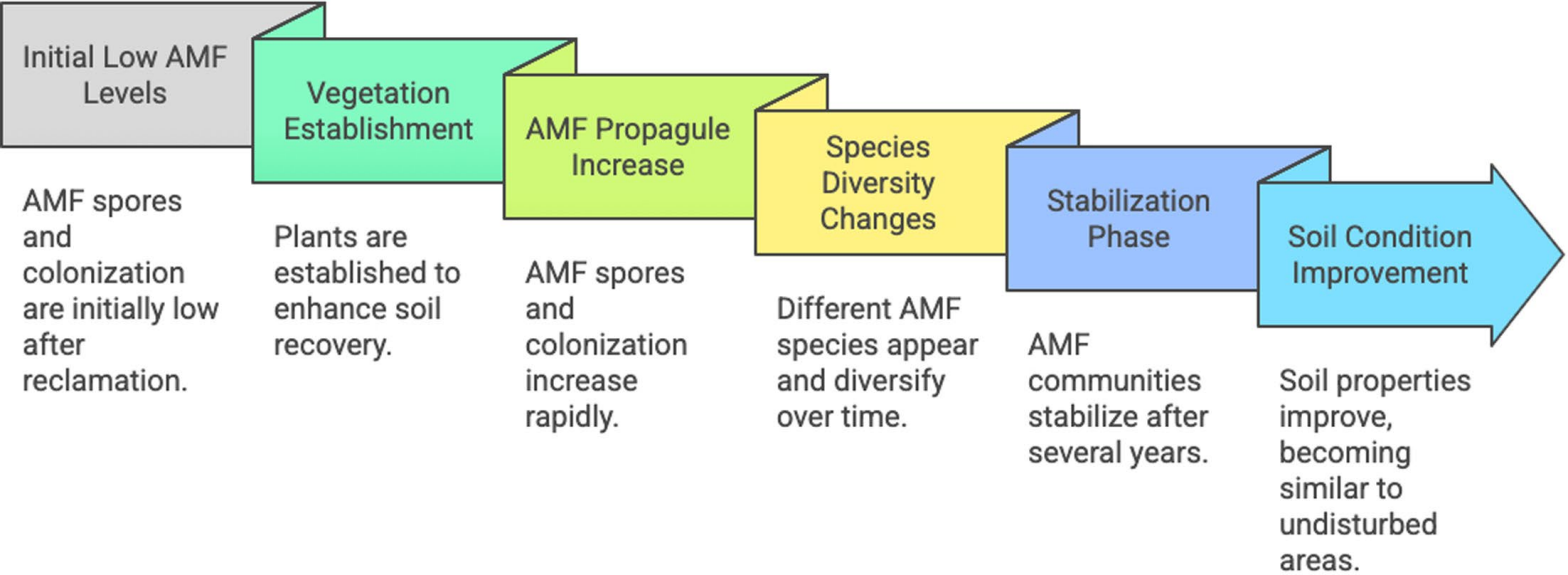
Schematic representation of the time-course of arbuscular mycorrizal fungi in mine restoration. Created by Napkin.

Thus, during restoration of mining environments, successive AMF species and increasing diversity and propagules number generally follow herbaceous in early plant successions and soil reconstruction, with positive impacts observed on biomasses and survival rates, lowering the translocation of toxic elements from roots to shoots (Figure 2). AMF inoculation seems not always efficient, although positive results have been obtained under tropical climates.

### 3.2 Forest environments, AMF, and EMF communities in mine restoration

Under the coal mining environment in Australia, Ngugi et al. (2019) characterized the diversity of soil fungal communities along a restoration chronosequence from 3 to 23 years. Fungal richness on rehabilitated sites was significantly larger than on non-mined sites, suggesting that mixing of topsoil during stockpiling resulted in a composite microbial community. Fungal community composition was significantly influenced by edaphic variables and the length of rehabilitation, with mined sites becoming more similar to non-mined sites over time. Fungal populations associated with EM were relatively more abundant than those associated with AM and declined in response to disturbance but recovered over time on the woody-dominated sites, indicating a strong coupling of these fungi with aboveground vegetation.

McMahen et al. (2022), using primary successional mine reclamation materials with or without forest soil additions, first conditioned the soil with *Salix scouleriana*, an early successional shrub with low mycorrhizal dependence or a later-successional *Picea* EM conifer. The same plant species and later-successional plants, *Picea* and/or *Thuja plicata*, were grown in conditioned or unconditioned control soils. They found negative intraspecific soil legacies for *Salix*, with pathogen accumulation, but neutral to positive intraspecific legacies for *Picea*, with increased mycorrhizal fungal colonization and diversity, confirming that soil legacy effects vary with plant nutrient acquisition strategy. Soil legacy effects of *Salix* on next-stage successional species (*Picea* and *Thuja*) were negative, potentially due to allelopathy, while EM *Picea* had neutral to negative legacy effects on AM *Thuja*, likely due to incompatible mycorrhizae. Thus, positive biological legacies may be limited to scenarios where mycorrhizal-dependent plants grow in soil containing legacies of compatible mycorrhizae, indicating that initial mine restoration actions may potentially exert long-term effects on plant community composition, even in primary successional soils with low microbial activity.

In Eastern Germany, Hüttl and Weber (2001) reviewed the forest ecosystem development after rehabilitating open-cast lignite mines generating spoil acidified and phytotoxic dumps. *Pinus* stands on mine sites for approximately 35 years did not show differences with stands on non-mined sites of the region; thus, natural forest succession seems applicable, with modification, at least for the early stages of forest development. Soil organism abundance and activity at mine sites had already reached levels typical of non-mined sites after approximately 20–30 years. However, mine soils are very different from non-mined soils of the region, chemical development being dominated by processes originating from pyrite oxidation and geogenic, i.e., lignitic, SOC substituting for some functions of pedogenic SOM. Rooting was hampered but not completely impeded in strongly acidified soils, and roots and mycorrhizae were apparently able to make use of the heterogeneity of young mining soils.

Through the restoration of an iron tailings deposit in China, Zhu et al. (2022) observed that the presence of vegetation significantly improved most soil properties and that, compared to *Pinus koraiensis*, *Populus simonii*, and *Robinia pseudoacacia* tree species and their associated herbaceous vegetation could better improve soil total C, total N, total P, and available P. In addition, soil EMF communities significantly differed depending on different revegetation types, and EMF diversity in *Robinia* was greater than in *Pinus* and *Populus* trees, suggesting that *R. pseudoacacia*, together with its additional advantage of N_2_ fixing symbiosis, could be a suitable species for the revegetation of those iron mine tailings.

Moreover, in Ni and Co mining environments in Madagascar and New Caledonia, plants usable as facilitators in ecological restoration were identified by characterizing ectomycorrhizal communities. In Madagascar, while four ectomycorrhizal tree species locally dominate the canopy which shares most of their ectomycorrhizal partners, only *Asteropeia mcphersonii* spontaneously regenerated following original ecosystem destruction, making this species a candidate for its use as facilitator (Henry et al., 2015). In New Caledonia, heavy metal toxicities (Ni, Co, Mn, and Cr), Mg excess, nutrient paucity, and >90% iron oxide excess enable only a few planted species to grow after ecosystem destruction, among which *Acacia spirorbis*, by its high capacity to form EM symbioses with a large spectrum of fungi regardless soil categories (Houles et al., 2018), seems a good facilitator candidate.

Thus, contrary to the more adaptable initial AM herbaceous species, the successful reinstallation of forest ecosystems in mine restoration areas seems to depend on specific successional tree species and associated AM and EM mycorrhizal fungi adapted to the local climate and soil conditions. See Table 1 for a synthesis of results cited in Sections 2 and 3.

## 4 AMF, plants, and soil microbiome in ecological restoration

The diversity of soil microbiota, such as bacteria, fungi, and microfauna associated with them, is crucial for understanding the ecological complexities between plants, microbes, soils, and climates and their roles in ecological restoration. In addition to AMF, soils also contain billions of microorganisms that mineralize the SOM and metabolize other molecules, some of them directly associated with plants and AMF, such as plant growth-promoting rhizobacteria, including free-living and symbiotic N-fixers and mycorrhiza-helper bacteria (Table 2).

### 4.1 AMF, plants, and soil microbiome in metal-contaminated soils

Khan (2006) reviewed the ecological complexity and diversity of plant–microbe–soil combinations and the role of AMF in phytorestoration of heavy metal (HM) contaminated soils, i.e., mycorrhizoremediation. To exploit microbes as biofertilizers, biostimulants, and bioprotectants against pathogens and heavy metals, the ecological complexity of microbes in the mycorrhizosphere needs to be taken into consideration with optimization of rhizosphere/mycorrhizosphere systems, the help of multidisciplinary investigations using molecular, biochemical, and physiological techniques and greater collaborative efforts between biologists, soil chemists, and physicists (Smith, 2002). Jha and Songachan (2022) further emphasized the importance of understanding the ecological dynamics of different plants, microbes, soils, and climates, as well as their role in the phytoremediation of polluted soils, by analyzing plant roots and a variety of soil microbiota, such as related bacteria, fungi, and microfauna. Indeed, while the role of AMF in the phytoremediation of soils contaminated by HM, radionuclides, polycyclic aromatic hydrocarbons, and other contaminants is now obvious, the biological dynamics of plant–microbe–soil interactions need to be further investigated as consortia. Moreover, Raklami et al. (2022) reviewed the plant–microorganism-based phytoremediation techniques through the interactions between plants, their associated microbiomes, environment, and biological processes and then discussed how they shape the assembly of plant-associated microbial communities and modulate metal(loid) remediation, providing insights into the underlying remediation strategy mechanisms, key challenges, and future directions for bioremediation of metal-polluted agricultural soils.

The regulation mechanisms of plant response to HM stress mediated by endophytic fungi, including AMF, dark septate endophytes (DSE), and plant growth-promoting endophytic fungi, were further reviewed by Zheng et al. (2023). These mechanisms involve a coevolution strategy to improve the ability of plants to adapt to HM stress. They can increase the synthesis and maintain the balance of host hormones, strengthen osmotic regulation, regulate C and N metabolism, and increase immune activity, antioxidant enzyme, and glutathione activity. They also help to improve the detoxification transport and HM emission capacity of the host by producing iron carrier, metallothionein, and 1-aminocyclopropane-1-carboxylic acid deaminase such that the combination of endophytic fungi and hyperaccumulation plants provides a promising technology for the ecological restoration of HM contaminated soil.

In a metal-contaminated mine waste revegetation project in Montana, US, Moynahan et al. (2002) measured the microbial community structure and C-utilization diversity in different restoration treatments, including two controls of raw tailings, tilled or not. They found that heterotrophic bacteria were significantly higher in the limed and topsoil treatment than in controls, that actinomycetes and fungi increased in the tilled control, and that endospores were significantly higher under topsoil addition and undisturbed plots. They also observed that C-utilization activity was very low in untreated plots, intermediate in lime-treated plots, and very high in topsoil and undisturbed plots. AM colonization levels of two grass species showed low levels of colonization in control, shallow-limed, and lime slurry-injected plots and high levels on the deep-limed and topsoil-addition plot, showing the specificities of soil physical treatments and additions to restore different categories of soil microorganisms.

Moreover, specific relationships between AMF, host plants, and other soil microbes were studied along a gradient of HM contamination at a site of Zn smelters under restoration in Pennsylvania, US (Glassman and Casper, 2012). The addition of a non-AMF soil microbial wash of either origin increased the efficiency of AMF from low-contaminated (LC) soils. However, it decreased the efficacy of AMF from high-contaminated (HC) soils in promoting plant growth of the C4 grass *Sorghastrum nutans*, which showed high mortality in HC soil, whereas plant biomass of the native C3 grass *Deschampsia flexuosa* did not vary with AMF source and/or the microbial wash treatment. While AMF origin did not affect root colonization of *D. flexuosa* by AMF, the presence and origin of AMF affected the number of non-mycorrhizal (NMF) morphotypes and root colonization, and the addition of non-AMF soil biota reduced Zn concentrations in shoots of *D. flexuosa*. Thus, the non-AMF biotic context affected HM sequestration and associated NMF in *D. flexuosa*, and it interacted with AMF to affect plant biomass in *S. nutans*, showing the species specificity of AMF–plant–microbe interactions.

Microbial response was further explored by Hinojosa et al. (2005) in a mine acid-metal spill in Spain by comparison between PLFAs and direct soil extraction and transesterification of total ELFA methods. Inferences from the whole community–diversity analysis and correlations of individual fatty acids with metals suggested that Cu, Cd, Zn, and pH were the most important in affecting microbial community structure. The microbial stress marker, monounsat fatty acids, was significantly lower for reclaimed and polluted soil over non-polluted soils for both PLFA and ELFA extraction. Another stress marker, the monounsat/sat fatty acids, only showed this for the PLFA. The general fungal marker, the AM marker, and iso-and anteiso-branched PLFAs (Gram + bacteria) were suppressed with increasing pollution, whereas Gram-bacteria increased with metal pollution. For both extraction methods, richness and diversity were greater in non-polluted soils and lowest in polluted soils, and the ELFA method was sensitive to reflecting metal pollution on microbial communities.

In the proximity of a Pb/Zn smelter under a subtropical climate in China, Zhang et al. (2006) also explored the microbial community structure and function in revegetated industrial barren and adjacent natural areas. The revegetation did not significantly change PLFAs and enzyme profiles with time, while Gram-bacteria, AM fungi, total fungi, actinomycetes, algae, protozoa, fungi/bacteria, monounsat/sat fatty acids, and enzyme activities including protease, CM-cellulase, and β-glucosidase consistently increased with time after revegetation, corresponding to the increase in total N, pH, and porosity in the revegetated soils. Moreover, cyclopropyl fatty acids/monoenoic precursors significantly decreased after revegetation and were inversely correlated with the above soil parameters. Thus, based on either the PLFA or enzyme activity, the revegetated sites clustered separately from the control with time after revegetation and the microbial community structure was closely linked to its function during the revegetation, and soil physico-chemical parameters, except P and WHC, are more important factors in determining the structure and function of soil microbial community than HM contents. The native species *Paulownia fortunei* used for revegetation considerably improved the structure and function of the soil microbial community and was thus effective for remediation of those industrial HM-contaminated barrens.

In dolomite-amended polymetallic contaminated tailings in Arizona, US, Creamer et al. (2023) further evaluated whether the planting of native perennial grass with a consortium of diazotrophic microbial endophytes and municipal waste compost—alone and in combination—enhanced plant growth while stabilizing metal(loids). They found that, although most of the added endophytes were not uniquely identified, the best plant growth and fertility outcomes were achieved with a combination of dolomite to reduce acidity, compost to increase N, and a mixed consortium of seed-coated endophytes to synergistically increase organic C and grass biomass yields. Compost reduced shoot and root concentrations—but not yields—of metal contaminants. The endophytes increased foliar Cd, Co, Mn, and Pb and mobilized Pb and Zn from the tailings. Root stabilization of Cd, Co, and Mn did not require amendments. Thus, due to potential PO_4_ solubilization and siderophore production by the endophyte consortium, strategies to capture solubilized metal(loids) may be needed for sulfidic tailings with metal(loids) associated with mobile mineral phases.

In ultramafic (i.e., serpentine) soils in Albania, Lopez et al. (2019) further explored the community diversity and potential functions of rhizosphere-associated bacteria of Ni hyperaccumulating plants from four widespread species: *Noccaea ochroleuca*, *Odontarrhena smolikana*, *O. rigida*, and *O. chalcidica*. They showed that Proteobacteria, Actinobacteria, and Acidobacteria dominated the soil bacteria while only the Proteobacteria group was relatively abundant and underlined the influence of CEC on the bacterial community’s diversity and structure. Based on the predicted metagenomes, the genes belonging to amino acid, lipid, and carbohydrate metabolisms were identified as major gene families. Moreover, in New Caledonian serpentine ecosystems, Amir et al. (2022) reviewed that AMF are abundant and concerned nearly all plant species, including Ni-hyperaccumulator plants and sedges, generally considered non-mycorrhizal, the adaptation of AMF to the extreme conditions of these soils leading to high levels of metal tolerance and noticeable originality of the taxa. Combinations of AMF isolates with complementary functional traits showed highly synergistic effects on various plant species, and the present studies mainly focus on the additive effects of AMF and mycorrhiza-helper bacteria.

Ten years after a Fe mine exploitation in Brazil, Cardoso et al. (2020) explored the diversity of N-fixing microorganisms and Actinobacteria in the deactivated mining site where the revegetation process was begun (RV) and a reference site with natural vegetation (NT) at proximity. In both sites, the most abundant archaeal and bacterial groups included Euryarchaeota, Thaumarchaeota, Proteobacteria, Actinobacteria, Acidobacteria, and Verrucomicrobia. Proteobacteria and Actinobacteria were most abundant in RV sites, while Acidobacteria and Verrucomicrobia were most abundant in NT sites but the majority of identified bacterial genera were shared by RV and NT, with only less abundant phyla specifically found in NT or RV. Soil P, pH, and particle density were significant in RV, while Fe, Ca, SOM, potential acidity, and dispersed clays were significant in NT, showing differences in soil characteristics that led to the prokaryotic composition. DGGE profiles of N-fixing microorganisms revealed their predominance in both sites, while after 10 years, prokaryote diversity increased in the RV site, indicating the RV soil resilience.

In gold mining waste rock dump rehabilitation in Burkina Faso, Yonli et al. (2022) tested the association of microbial and/or manure amendments with *Senegalia senegal* seedlings, a multipurpose tree colonizing Sub-Saharan mining sites, by inoculation with a native isolate of the AMF *Rhizophagus aggregatus*, the commercial isolate of *R. irregularis*, and a *Mesorhizobium plurifarium* strain with three substrate types then out-plantation on the waste rock. Under nursery conditions, manure-enriched substrates showed less nodulation and AM colonization but increased plant growth compared to un-amended substrate; inoculation did not increase AM colonization and plant growth, and *R. aggregatus*, alone or with *R. irregularis* or *M. plurifarium*, showed the highest plant growth. On un-amended substrates, inoculation with *R. aggregatus* + *M. plurifarium* or *R. irregularis* significantly enhanced root colonization rates without altering plant growth. In field conditions, plant growth and survival were reduced under high-rate manure amendments, likely due to less AM colonization and root nodulation observed in the nursery, but strongly colonized plants on the un-amended and moderately amended substrate showed greater survival after outplanting, suggesting that microbial inoculation and moderate levels of manure are a viable option for this mining site rehabilitation with *S. senegal*.

In extreme tailing dumps, such as abandoned antimony tailings, Xiao et al. (2019) explored the distribution pattern of root-associated bacteria and their responses to environmental factors in the native plant *Bidens bipinnata*, growing on both an Sb tailing dump (WKA) and adjacent normal soils (WKC). They found that the rhizosphere microbial diversity in the tailing dump was significantly different from that in the adjacent soil and that such variation was significantly related to soil nutrients (TC, TOC, and TN) and metal(loid) concentrations (Sb and As). Some dominant genera were significantly enriched in WKA, suggesting their adaptation to harsh environments and their involvement in nutrient and metal(liod) cycling, such as N-fixing (*Devosia*, *Cellvibrio*, *Lysobacter*, and *Cohnella*), P-solubilizing (*Flavobacterium*), and Sb-and As-oxidating (*Paenibacillus*, *Bacillus*, *Pseudomonas*, and *Thiobacillus*), those specific root-associated bacteria, governed by soil edaphic factors, playing important ecological roles for the successful colonization of *B. bipinnata* in this tailing dump.

Finally, García-Sánchez et al. (2019) evaluated the implication of combining a mycoremediated dry olive residue (MDOR) amendment inoculated either with *Penicillium chrysogenum* or with *Chondrosteum purpureum* and the inoculation of the AMF *Funneliformis mosseae* under 30-and 60-day mesocosm experiment. Changes in the soil labile organic C and N fractions were observed throughout the experiment, as well as increases in the abundance of PFLAs for bacteria, actinobacteria, and Gram− and Gram+ bacteria at the end of the experiment. MDOR amendments boosted fungal and AMF communities, and AMF root and soil colonization were enhanced by adding MDOR and *F. mosseae*, showing that the composition and functionality of microbial communities is an important ecological indicator of the functional restoration of this metal-polluted soil and suggesting the suitability of the combined MDOR and AMF treatment as a reclamation practice.

**Figure 3:**
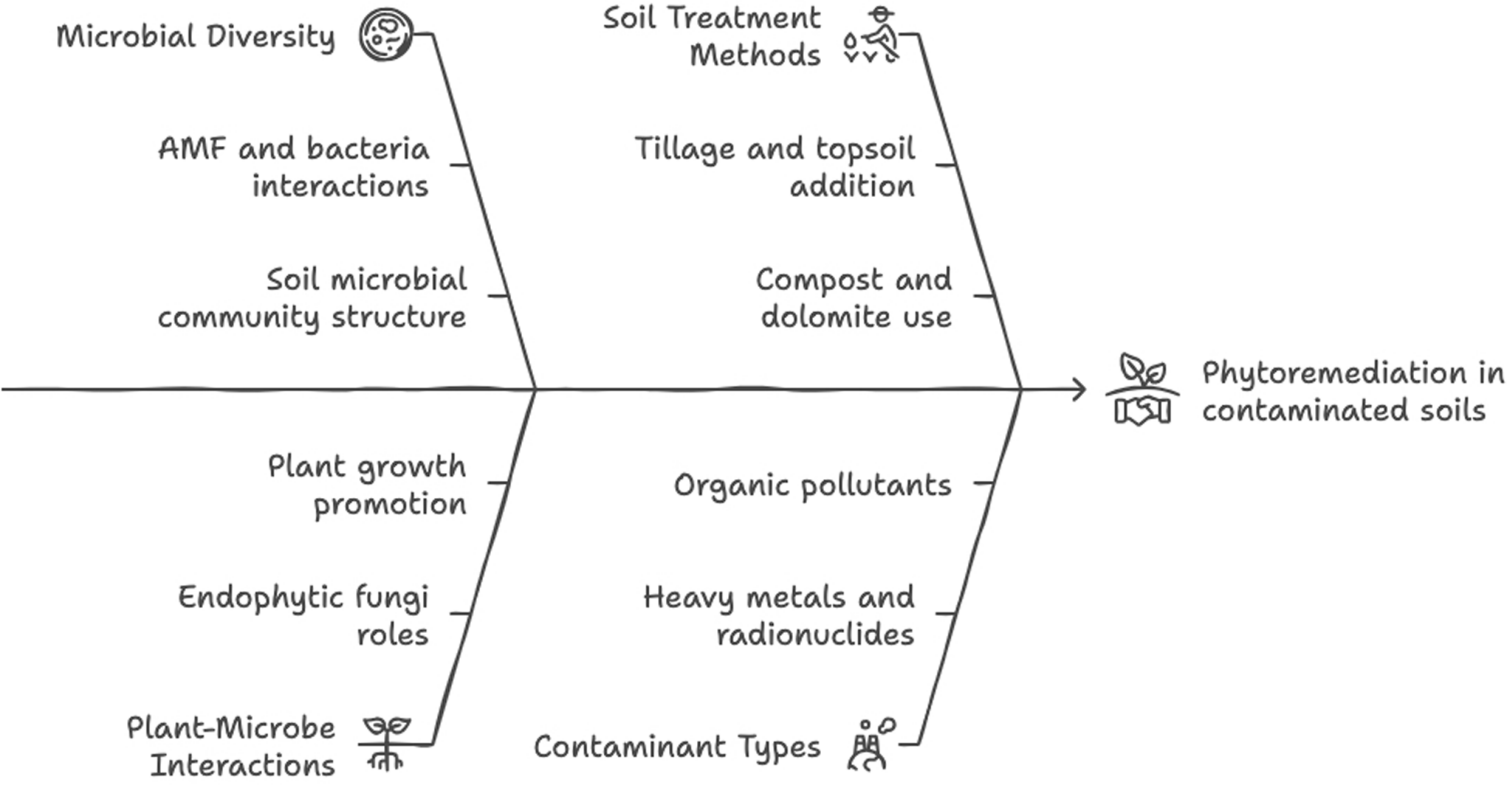
Schematic representation of the microbial interactions in enhancing phytoremediation. Created by Napkin.

Taken together, previous studies on rehabilitation of metal-polluted sites showed the great species specificities of plant–soil–microbe interactions and relationships with each soil mineral cycle and function (Table 2 and Figure 3), which prevent any generalization of those complex phenomena.

### 4.2 AMF, plant, and soil microbiome in other ecosystem restoration and standardization of parameters

In coal mining waste rehabilitation in China, Liu et al. (2019) observed higher plant species richness, canopy coverage, and bacterial community diversity in artificially planted areas but low similarity with naturally regenerated areas, with soil pH, moisture content, total C, SOM, N, and bulk density having a greater impact on soil bacterial community structure and diversity. Moreover, the effects of an AMF strain of *Glomus mosseae* and phosphate solubilizing bacteria (PSB) on phytate mineralization and subsequent transfer to the host plant *Medicago sativa* were investigated in two-compartment microcosms, showing higher available P, above and underground biomass in combined inoculation of AMF-PSB than other treatments in root and hyphae compartment (Bi et al., 2019). As AMF or PSB inocula had a significant influence on soil acid phosphatase (ACP) activities, AMF-PSB enhanced phytate mineralization and improved plant biomass, and P and ACP increased the shoot and root biomasses; this microbial consortium is proposed to be used as bioinoculant to enhance plant sustainable production in abandoned solid waste of coal mine.

In petroleum-contaminated soils, Dagher et al. (2020) further tested whether repeated inoculations with a consortium of 10 isolates of Proteobacteria influenced plant productivity and the AMF assemblages associated with the root and rhizosphere of four plant species growing in non-contaminated natural soil vs. contaminated sediments. Their results showed that while inoculation caused a significant shift in AMF communities, substrate contamination had a much stronger influence on their structure. Inoculation significantly increased plant biomass and was associated with decreased petroleum hydrocarbons, confirming the dominance of soil chemical properties over biotic factors and inputs, such as plant species and microbial inoculations, in determining the plant-associated AMF communities. The biochemistry of AMF detoxification of hydrocarbon-contaminated soils was explored by Calonne et al. (2014), which proved that the exposition of *R. irregularis* to a polycyclic aromatic hydrocarbon involved the fungal triacylglycerol metabolism, providing carbon skeletons and energy necessary for membrane regeneration and/or for hydrocarbon translocation and degradation, and activating the phosphatidic acid and hexose metabolisms.

In a Mediterranean salt marsh in Southeast Spain, Caravaca et al. (2005) tested whether a spatial salinity gradient can affect the microbiological and biochemical properties of the rhizosphere soil of *Inula crithmoides* and the AM root colonization. They observed no significant differences in the soluble C and carbohydrates fractions or the microbial biomass C values in the rhizosphere soil of *I. crithmoides* among the different zones of the salt marsh, whereas dehydrogenase activity, hydrolases (urease, protease, phosphatase, and-glucosidase), and AM colonization in *I. crithmoides* roots were greater in the higher salt marsh zones, corresponding to those of lower rhizosphere soil salinity, showing that soil salinity and AMF colonization were inversely associated with previous soil microbial activity parameters. Bioactive SOM fractions that determine soil productivity, however, were not related to soil salinity.

More generally, in coastal ecosystems, Farrer et al. (2022) reviewed the ecology of plant–microbial symbioses and their potential use in restoration, including mycorrhizae, N fixers, endophytes, rhizosphere microbes, and pathogens, focusing on four common coastal communities: sand dunes, marshes, mangroves, and forests/shrublands. They conclude that all those microbial symbionts are largely responsible for the health of coastal plants by affecting plant establishment, growth, competitive ability, and stress tolerance, as well as modulating biogeochemical cycling, encouraging the use of microbial symbionts to augment the restoration success of these ecosystems.

For evaluating the success of ecological reclamations, Domínguez-Haydar et al. (2019) tested the suitability of biological, chemical, and physical quality indicators–and their combination in a General Indicator of Soil Quality (GISQ)—to monitor soil quality in restored areas of an open cast coal mine. Their biological indicator showed a significant recovery of soil invertebrate communities along the chronosequence. Taxonomic richness increased from 7 taxa in the 1st year to 13–17 taxa in 6–20-year sites, far less than in forests (19–20). Soil pH, bulk density, and proportion of physical and macro-aggregates were highest in the 1-year site, while SOM, total N, and biogenic aggregates were highest in older sites. In general, the three sub-indicators and the GISQ yielded the lowest values in the 1-year site (from 0.1 to 0.3 on a scale of 0.1–1.0), intermediate in the 16- and 20-year sites (0.4–0.7), and highest values in the two forests (0.4–1.0), proving that the GISQ method was efficient in assessing progress in the reclamation process.

Gastauer et al. (2020) further constated the great challenge of comparing rehabilitated sites with target ecosystems, as well as integrating individual environmental and eventually collinear variables into a single tractable measure of the state of a system before effective rehabilitation indicators may be modeled. Since a consensus is lacking regarding which and how many variables need to be surveyed for a reliable estimation of rehabilitation status, they proposed a multivariate ordination to integrate variables related to ecological processes, vegetation structure, and community diversity into a single estimation of rehabilitation status, from a curated set of 32 environmental variables retrieved from non-revegetated, rehabilitated, and reference sites associated with iron ore mines in Brazil. Their multivariate approach was able to adequately address collinearity among variables, identify biases toward single variables, surveys, or analyses, and identify the minimum number of environmental variables necessary to achieve reliable estimations of the rehabilitation status, proposing the definition of case-specific environmental indicators for more rapid assessments of mineland rehabilitation.

## 5 General conclusion

At the end of this temptative to compare the main reviews and some specific studies about the roles of mycorrhizal symbioses and associated soil microbiomes in several areas of ecological restoration, there is a general need to better define the essential environmental parameters to be able to compare the diverse studies. In particular, the time of observation after rehabilitation operations is necessarily various but often difficult to retrieve and rarely mention in abstracts. Moreover, each component of the trilogy plant–soil–microbiomes is variously considered according to the different points of view and researchers’ specializations, although these three components are of the same importance in the comprehensiveness of ecosystem dynamics, which emphasizes the need for more collaborative multidisciplinary works.

Plant communities, either exotic or native, reintroduced or naturally occurring since the beginning of the ecosystem restoration or through plant successions, are always the drivers of belowground biodiversity. Indeed, since each vegetal species is associated with a specific belowground microbiome, the more varied above-ground diversity ensures the diversity of the belowground life.

AMF species are changing with time post-revegetation and plant communities, with larger spore species of *Gigaspora* and *Scutellospora* appearing lately in plant successions compared to smaller spore species belonging to Glomaceae and related families. These AMF species successions seem essential since even when late plant species are reintroduced, the efficacious AMF species often remain the early seral ones. Nevertheless, the time course and variations of AMF diversity during the years following restoration seem very specific to each particular context. Introduced exotic plant species induced variation of AMF communities as compared to non-restored sites or native species, but AM symbiosis always improves plant and soil characteristics. The benefits of AMF inoculation with either native or commercial exotic strains are diverse, according to each environmental context.

Various specific and very dynamic interactions occur between AMF and soil microbiome with various consequences on soil structure, plant physiology, and nutrient status according to each environmental context. Proteobacteria, Actinobacteria, and Acidobacteria generally represent the more active groups associated with rhizosphere functions. Generalizations are often impossible, apart from micro-ecosystem plant–soil–microbes specificities, given the fact that soil microbes are also greatly adaptable and interchangeable, which signifies that several soil functions can be achieved by several species or groups of microorganisms, depending on each specific microbiome at each geographical and time point, such that the «soil picture» is constantly evolving and adapting to each micro-environmental change. If an exhaustive understanding of those complex plant–soil–microbes relationships seems quasi-impossible, further integration of the closely interlinked soils’ biological and physico-chemical parameters for each ecosystem context will permit a closer approach to it.
